# Direct fluorescent *S*-genotyping reveals genetic diversity and pedigree inconsistencies in red-fleshed apple hybrids and American heritage varieties

**DOI:** 10.1007/s00425-025-04918-4

**Published:** 2026-01-06

**Authors:** Gergő Szendy, Dávid Polgári, Magdolna Tóth, Attila Hegedűs, Júlia Halász

**Affiliations:** 1 Horticultural Plant Genetics Group, Department of Plant Biotechnology, Institute of Genetics and Biotechnology, Hungarian University of Agriculture and Life Sciences, 1118 Budapest, Hungary; 2 Agricultural Institute, Centre for Agricultural Research, Martonvásár, Hungary; 3 Department of Agronomy, Institute of Agronomy, Hungarian University of Agriculture and Life Sciences, 2100 Gödöllő, Hungary; 4 Almakúti Ltd., 8353 Zalaszántó, Hungary

**Keywords:** Red-fleshed apple, *S*-allele, *S*-genotyping, *Malus* × *domestica*, Marker-assisted selection

## Abstract

Main conclusion A PCR-only *S*-genotyping was optimized for red-fleshed apple hybrids carrying the R_6_ allele of *MdMYB10*. Sixteen distinct *S*-alleles were identified. New hybrids show unique *S*-allele frequencies, enhancing breeding diversity. Red-fleshed apples are increasingly valued for their esthetic appeal and potential health benefits, making them attractive targets in modern breeding programs. In this study, 80 novel red-fleshed and six white-fleshed apple hybrids, along with their parental cultivars, were genotyped for the R_6_ allele of the *MdMYB10* gene and for their *S*-alleles to assess pollination compatibility. All red-fleshed hybrids carried the R_6_ allele, confirming their type I red-fleshed phenotype. A previously published high-throughput *S*-genotyping protocol was optimized to achieve single base-pair accuracy, enabling a streamlined, PCR-only workflow without the need for restriction enzyme digestion. 16 distinct *S*-alleles were identified, and allele-specific primers and sequencing were used to verify ambiguous cases. The genotyping results revealed inconsistencies in pedigree records and pollination procedures, underscoring the importance of molecular validation in breeding programs. Additionally, two American heritage apple cultivars were found to carry three *S*-alleles despite being diploid, suggesting possible segmental duplications. The *S*-allele frequency distribution in the new hybrids differed from traditional Hungarian and international germplasm, indicating their potential to broaden the genetic base of disease-resistant apple breeding. This study provides a refined genotyping approach and valuable insights into the genetic composition of novel apple hybrids, contributing to improved breeding strategies and germplasm management.

**Supplementary Information:**

The online version contains supplementary material available at 10.1007/s00425-025-04918-4.

## Introduction

The apple is the third most popular fruit cultivated worldwide behind watermelons and bananas (FAOSTAT 2023). Besides fresh consumption, the fruit can be processed into juice, cider, purees, chips, and beauty products (Matsumoto 2014). While over 30,000 apple varieties are known, some 7,500 of these named and many cultivars are released every year (Volk et al. 2015; Sheick et al. 2020; Górnaś et al. 2022; Szot et al. 2022;). Produce of the competitors and changing demand from customers continuously compel growers to plant new cultivars in order to stay ahead of this curve (Legun 2015).

With annual sales worth billions of dollars, the apple industry relies on consistent yields, which fundamentally depend on successful fertilization. Although some accessions display parthenocarpy (Wu et al. 2024), commercially grown diploid cultivars are self-incompatible and require a pollinizer cultivar for cross-pollination to ensure economically adequate fruit set and, ultimately, profitability (Crane and Lawrence 1928; Ramírez and Davenport 2013). Sexual incompatibility between the cultivars planted in an orchard can lead to inadequate fruit set (Jackson 2003) while partial compatibility can result in less than desirable fruit set and deformed fruits due to inadequate seed set (De Witte et al. 1995; Goldway et al. 1999; Long et al. 2010).

Compatibility studies initially consisted of phenotypic incompatibility test (Kobel et al. 1939) and controlled pollination experiments with pollen tube growth tests (Modlibowska 1945) to infer the incompatibility alleles while modern methods use allele-specific PCR (Broothaerts 2003; Long et al. 2010; De Franceschi et al. 2018; Abdallah et al. 2024), CAPS (Kim et al. 2009), PCR with universal primers (Larsen et al. 2016), qPCR (López-Girona et al. 2021), and mini-sequencing (Kasajima et al. 2017), or a combination of these approaches in order to provide accurate results. Apple possesses a gametophytic self-incompatibility system, like all other plants in Rosaceae, which operates on a non-self-recognition basis. The multi-allelic *S*-locus encodes self-incompatibility ribonucleases (*S*-RNases), which are produced in the style, and a group of F-box proteins (*S*-locus F-box Brothers, SFBBs), which accumulate in the pollen (Sassa et al. 2007). According to the current model of non-self-recognition, each SFB protein recognizes a specific set of non-self *S*-RNases and marks them for proteolytic degradation through the ubiquitin–proteasome pathway (Kubo et al. 2010; De Franceschi et al. 2018; Pratas et al. 2018). Due to the large number of *SFBB* genes in a single haplotype, *S*-genotyping methods focus on identifying the *S*-*RNase* genes in apple (López-Girona et al. 2021). The discovery of new *S*-alleles in both cultivated and wild apple genotypes along with the necessity of incorporating them into compatibility testing underscores the need for continuous methodological development (De Franceschi et al. 2018).

*S*-alleles are of paramount importance during breeding as well. Not only accessions with matching *S*-genotypes cannot be crossed with each other, if an economically important trait is closely linked to matching *S*-alleles then even semi-compatible crosses could become impractical (Umemura et al. 2013). *S*-alleles are also used when screening progenies to confirm the parental cultivars (Sakurai et al. 2000; Long et al. 2010) and it is also an effective tool to distinguish between accessions with identical names or very similar phenotypes in gene banks and germplasm collections (Halász et al. 2011).

Red-fleshed apples present a novel possibility for both breeders and growers. These apples not only have a striking appearance but can be also considered functional foods with stronger health benefits than regular apples (van Nocker et al. 2012; Wang et al. 2015). The red flesh can be the result of two distinct mutations; these are referred to as types I and II (Volz et al. 2007). Flesh color development in type I red-fleshed apples is controlled by the upregulation of the *MdMYB10* transcription factor (Espley et al. 2009); its expression level has been shown to directly correlate with the anthocyanin content in plant tissues (Chagné et al. 2013). The presence of a single repeat (R_1_) unit of 23 bp in the promoter of the *MdMYB10* gene results in a white-fleshed phenotype, while the insertion of five additional tandem repeats (R_6_) enhances the ability of *MdMYB10* to bind to its own promoter region and autoregulate its own expression. This mutant allele with six repeats confers the red-fleshed phenotype. Actual level of anthocyanin accumulation and thus flesh coloration differs among R_6_ red-fleshed plants and is heavily influenced by the activity of other transcription factors (Wang et al. 2018, 2024; Jiang et al. 2019).

This study aimed to screen 80 new red-fleshed and six white-fleshed hybrids, along with their parental cultivars, for the R_6_ allele of the *MdMYB10* gene. This allele is associated with increased anthocyanin accumulation. This study aimed to determine the *S*-genotype of the hybrids to evaluate their pollination compatibility with commercial cultivars and to optimize a previously published high-throughput *S*-genotyping method by improving its accuracy to single-base pair resolution, enabling a PCR-only workflow and reducing the number of required reactions.

## Materials and methods

### Plant material

In total, 107 apple cultivars and genotypes (80 new red-fleshed hybrids, six white-fleshed hybrids, five red-fleshed and six white-fleshed parental cultivars and ten American heritage apples) were sampled. Plant material of the new hybrids and parental cultivars were provided by a breeding program of Almakúti Ltd. (Zalaszántó, Hungary). The American heritage apples were sampled in a private orchard (Budapest, Hungary). It should be noted that the cultivar referred to as ʽPilot’ in this article is the American heirloom variety discovered in the early 1800 s (Downing and Downing 1872) and not the contemporary variety also called ʽPilot’, released by the Institut für Obstforschung Dresden-Pillnitz in 1988 (Fischer and Fischer 2004). In all cases, young leaves were collected 3–4 weeks after bud break in the spring and stored at − 20 °C until DNA extraction.

### DNA extraction and PCR analysis

Genomic DNA was extracted from healthy young leaves using DNeasy Plant Mini Kit (Qiagen, Hilden, Germany) and E.Z.N.A.^®^SP Plant DNA Kit (Omega Bio-tek, Norcross, GA, USA) according to the manufacturers’ specifications. To amplify *S*alleles, the following consensus primers were used: ASPF3-F (Kim et al. 2006), EIIWPN-R, S3/S5/S10-R, S16-R and S25-R together with the *S*_8_ allele-specific S8F and S8R primer pair (Larsen et al. 2016). The results were validated using 13 allele-specific primer pairs: FTC168-FTC169, OWB122-OWB123, FTC141-FTC142, FTC143-FTC144, FTC154-FTC155, FTC231-FCT232, FTC229-FTC230, FTC177-FTC226, FTC10-FTC11, FTC12-FTC228 (Broothaerts 2003), MdS_11_SpF-MdS_11_SpR, MdS_21_SpF-MdS_21_SpR, and MdS_44_SpF-MdS_44_SpR (Long et al. 2010). Detailed information on all primers used in this study is provided in Supplementary Table S1. For the analysis of *MdMYB10* alleles, the primers described by Espley et al. (2009) (GGAGGGGAATGAAGAAGAGG and TCCACAGAAGCAAACACTGAC) were used following the published protocol.

Reactions using DreamTaq™ polymerase (Thermo Fisher Scientific, Waltham, MA, USA) were performed in a total volume of 20 µl, containing: 40–80 ng DNA sample, 10 × DreamTaq™ Green buffer; 1.2 mM MgCl_2_, 0.15 mM dNTP, 0.4 μM forward and reverse primers, 0.65 U DreamTaq™ DNA polymerase, 3% DMSO, and 0.01 μg/μl BSA. Reactions using Phire Hot Start II polymerase (Thermo Fisher Scientific, Waltham, MA, USA) were performed in a total volume of 15 µl, containing: 10 µg of DNA, 5 × buffer, 200 µM of each dNTP, 0.5 µM of both primers, 0.3 µl of Phire Hot Start II DNA Polymerase, 3% DMSO, and sterile distilled water to complete the volume. Reactions using either Geneaid™ Hot Start Taq Master Mix with Dye (Geneaid Biotech Ltd, Taiwan) or Canvax HotBegan^™^ Hot Start Green Taq Master Mix (Canvax Reagents SL, Valladolid, Spain) were carried out according to the manufacturers’ recommended protocols. For fragment length detection, the ASPF3-F forward primer was labeled with the FAM fluorescent probe. Reactions were carried out in Swift Max Pro (ESCO Healthcare, Singapore) and Genesy 96 T (Tianlong, Xi’an, China) thermocyclers. PCR cycling conditions for each primer pair are summarized in Supplementary Table S2.

### Fragment length determination

PCR products were first visualized on a 1% TBE agarose gel (15–20 min at 80 V) under UV light after staining with ethidium bromide. For accurate size determination, the forward primer ASPF3-F was labeled with FAM fluorescent dye for the ABI PRISM 3100 Genetic Analyzer (Applied Biosystems, Foster City, CA, USA) automated DNA sequencer. The results obtained were evaluated using the Peak Scanner 3.0 software (Applied Biosystems Sizing Analysis Module) with a GS500 LIZ (Applied Biosystems) size standard.

### Cloning and sequencing

Cloning of PCR products was carried out using the pTZ57R/T vector (Thermo Fisher Scientific, Waltham, MA, USA). The ligated plasmid vectors were transformed into JM109 *Escherichia coli* competent cells (Zymo Research, Irvine, CA, USA). The size of the cloned sequences was determined on agarose gels using M13 primers. The plasmid DNA fragments were purified using the EZ-10 Spin Column Plasmid DNA kit (Bio Basic, Markham, ON, Canada) and then sequenced at least three repetitions in the forward direction using the M13 sequencing primers in an ABI PRISM 3100 Genetic Analyzer (Thermo Fisher Scientific, Waltham, MA, USA) automated sequencer. Homology searches were carried out using the Basic Local Alignment Search Tool (BLAST) provided by the National Center for Biotechnology Information (NCBI, Bethesda, MD, USA) (Altschul et al. 1990).

### Ploidy analysis

The ploidy levels for the American heritage apples were determined by flow cytometry using a Sysmex CyFlow Space Flow Cytometer (Sysmex Partec GMBH, Görlitz, Germany) equipped with a 365 nm wavelength UV LED diode. For nuclei isolation and staining, because of the high polyphenol content of apple tree leaves, a one-step CyStain UV OxProtect kit (Sysmex Partec GMBH, Görlitz, Germany) was used according to the manufacturer’s recommended protocol. Young leaves were freshly collected for the measurements, and approximately 1 cm^2^ of fresh leaf segments were chopped for one minute with a sharp razor blade in a 50 mm diameter plastic Petri dish after adding 500 µL of CyStain UV OxProtect Staining Solution (Sysmex Partec GMBH, Görlitz, Germany). The shredded leaves were incubated for one minute in the dish, and then the liquid fraction was transferred to the sample tube through a 50 µm CellTrics filter (Sysmex Partec GMBH, Görlitz, Germany). The suspension was washed into the sample tube with an additional 2 mL of CyStain UV OxProtect Staining Solution. After a five-minute incubation, the DNA content (> 10.000 nuclei/sample) was measured using UV LED diode illumination. Relative genome size was determined using Flomax 2.11 software (QA GmbH, Münster, Germany). The G_0_/G_1_ and G_2_/M peaks were determined by peak analysis method. Ploidy of ʽWaltana’, ʽPink Parfait’ and the other heritage apples were assessed by comparing the measured G_0_/G_1_ genome sizes to the diploid ʽKing David’ and the triploid ʽJonagold’ control samples.

### Evaluation of fruit flesh color intensity

**Fig. 1:**
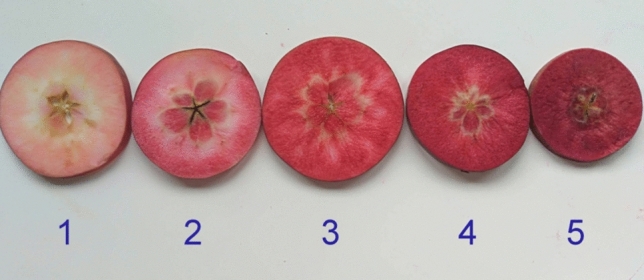
Classes of visually assessed red-flesh color intensity in apple, scored on a scale of 1 (faint pinkish tinge), 2 (moderate pink), 3 (pink-red), 4 (moderate red), and 5 (dark red)

The intensity of red coloration in apple fruit flesh was visually assessed using at least five representative fruits per accession and scored on a scale from 1 to 5, where 1 = faint pinkish tinge, 2 = moderate pink, 3 = pink-red, 4 = moderate red, and 5 = dark red; characteristic examples are shown in Fig. 1.

## Results

### *S*-allele detection of hybrids and cultivars

PCR amplification of the 86 new hybrids and the 11 parental cultivars using the ASPF3-F forward primer in combination with the EIIWPN-R reverse primer produced 11 distinct fragments smaller than 545 bp, and three additional fragments with the S3/S5/S10-R reverse primer (Table 1). In addition, ASPF3-F and EIIWPN-R amplicons larger than 1 kbp were also observed on agarose gels, corresponding to the *S*_3_-, *S*_5_-, and *S*_10_-alleles. These results were confirmed using the S3/S5/S10-R reverse primer, which specifically amplifies these alleles and yields distinct fragment sizes for each. The amplicons below 1 kbp were analyzed with capillary electrophoresis; all peaks were high, distinct, and appeared without stuttering for each sample (Table 1). The closest sizes were observed with the hybrid X149 in which the *S*_1_-and *S*_24_ -alleles resulted in fragment lengths of 545 bp and 540 bp, respectively, which were clearly visible as two distinct peaks on the electropherogram. The *S*_16_-allele was amplified in 12 red-fleshed hybrids and one new white-fleshed cultivar (ʽIsolda’) using the ASPF3-F forward and the S16-R reverse primers; the 447 bp size corresponded to the *S*_16b_-allele in all cases. The ASPF3-F forward and S25-R primer pairs did not amplify products in any of the samples; the *S*_8_-allele was detected only in one red-fleshed hybrid (X303) and one white-fleshed cultivar (ʽBellona’) using the allele-specific S8-F and S8-R primer pair. One allele amplified from *Malus purpurea* ʽRoyalty’ was detected as a 475 bp fragment that corresponds to the *S*_31_-allele.

**Table 1:** Detection of *S*-alleles using fragment size analysis determined by a capillary sequencer and PCRs with allele-specific primers

*S*-allele	Size (bp, in this study)	Size (bp, Larsen et al. 2016)	Allele-specific primers	Sequenced cultivar or hybrid	Accession number
ASPF3-F and EIIWPN-R					
*S*_1_	544	559–562	FTC168 FTC169	ʽWaltana’	PV693751
*S*_2_	350	370–372	OWB122 OWB123	–	–
*S*_6_	369	390–392	FTC141 FTC142	–	–
*S*_7_	320	340–342	FTC143 FTC144	–	–
*S*_9_	349	367–369	FTC154 FTC155	T184	PV693744
*S*_17_	369	390–392	FTC141 FTC142	ʽPilot’, ʽPink Parfait’, ʽRubaiyat’, ʽWaltana’	PV693747, PV693748, PV693749, PV693746
*S*_20_	516	535–537	FTC141 FTC142	–	–
*S*_24_	540	556–558	FTC231 FTC232	–	–
*S*_28_	371	392–394	FTC229 FTC230	–	–
*S*_31_	475	496–497	–	*Malus purpurea* ʽRoyalty’	PV693752
*S*_44_	375	396 (expected)	MdS_44_SpF MdS_44_SpR	–	–
ASPF3-F and S3/S5/S10-R					
*S*_3_	400	425–427	FTC177 FTC226	–	–
*S*_5_	378	401–403	FTC10 FTC11	ʽKatherine’	PV693750
*S*_10_	360	382–383	FTC12 FTC228	ʽWaltana’	PV693745
ASPF3-F and S16-R					
*S*_16b_	447	469–470	–	–	–

*S*-alleles for 10 American heritage apple varieties were also determined (Table 2). PCR reactions amplified two *S* alleles in ʽChestnut Crab’, ʽIngram’, ʽKatherine’, ʽKing David’, ʽMuscat de Venus’, ʽPilot’, ʽRubaiyat’ as well as ʽWickson Crab’ in using the ASPF3-F forward and EIIWPN-R and S3/S5/S10-R reverse primers, while in ʽPink Parfait’ (*S*_2_*S*_3_*S*_17_) and ʽWaltana’ (*S*_1_*S*_10_*S*_17_) three *S*-alleles were detected.

**Table 2:** *S*-allele constitution of American heritage varieties

Variety	*S*-allele	Verification method	*S*-allele	Verification method	*S*-allele	Verification method
Chestnut Crab	*S*_9_	FTC154 + FTC155 Sequencing	*S*_20_	FTC141 + FTC142	–	–
Ingram	*S*_1_	FTC168 + FTC169	*S*_9_	FTC154 + FTC155	–	–
Katherine	*S*_5_	FTC10 + FTC11, Sequencing	*S*_7_	FTC143 + FTC144	–	–
King David	*S*_1_	FTC168 + FTC169	*S*_24_	FTC231 + FTC232	–	–
Muscat de Venus	*S*_5_	FTC10 + FTC11	*S*_24_	FTC231 + FTC232	–	–
Pilot	*S*_7_	FTC143 + FTC144	*S*_17_	FTC141 + FTC142 Sequencing	–	–
Pink Parfait	*S*_2_	OWB122 + OWB123	*S*_3_	FTC177 + FTC226	*S*_17_	FTC141 + FTC142 Sequencing
Rubaiyat	*S*_3_	FTC177 + FTC226	*S*_17_	FTC141 + FTC142 Sequencing	–	–
Waltana	*S*_1_	FTC168 + FTC169 Sequencing	*S*_17_	FTC141 + FTC142 Sequencing	*S*_10_	FTC12 + FTC228 Sequencing
Wickson	*S*_3_	FTC177 + FTC226	*S*_5_	FTC10 + FTC11	–	–

### Verification of the results

Since the resulting fragments lacked the M13 tail used for fluorescent labeling by Larsen et al. (2016), the amplicon sizes were not directly comparable between the studies. For verification allele-specific primers (Broothaerts 2003; Long et al. 2010) were utilized for the *S*_1_–*S*_3_,* S*_5_,* S*_7_, *S*_9_, *S*_10_, *S*_6/17/20_, *S*_24_, *S*_28_, and *S*_44_ alleles. Amplification efficiency varied among polymerases. The *S*_7_ and *S*_9_ allele-specific products (FTC143–FTC144 and FTC154–FT155 primers) amplified weakly with Taq polymerase, whereas both alleles were strongly amplified using hot-start polymerase enzymes.

The FTC177 and FTC226 primers (Broothaerts 2003) amplifying the *S*_3_-allele did not work with the (C) PCR protocol, but using the modified (A) protocol detailed in Materials and Methods produced strong bands with light smearing in lanes where the *S*_*3*_ allele was present. The *S*_10_ allele-specific primers amplified not only the *S*_10_ but also the *S*_3_-allele, although somewhat weaker than the *S*_3_ allele-specific primers did. Therefore, the confirmation of the *S*_10_-allele required two PCR reactions with both the *S*_3_ and *S*_10_ allele-specific primers.

To verify the results for the heritage apple PCR products, allele-specific primers (Broothaerts 2003) were used, and nine amplicons were also cloned and sequenced (deposited in GenBank under accession numbers PV693744–PV693752) to confirm all detected alleles. Using PCR products from the ASPF3-F and EIIWPN-R reaction, the *S*_17_-allele was cloned from ʽPilot’, ʽPink Parfait’, ʽRubaiyat’ and ʽWaltana’ (PV693747, PV693748, PV693749 and PV693746, respectively). The fragment from ʽWaltana’ was 97.3% identical to the GenBank *S*_17_ sequence (MG598499) with 98.5% matching amino acids in the translated proteins while the other three fragments were 100% identical to the GenBank *S*_17_ sequence. In addition, the *S*_1_ (PV693751) and *S*_10_ (PV693745) alleles were cloned and sequenced from ʽWaltana’ and proved to be identical to the GenBank *S*_1_ (MG598487) and *S*_10_ (MG598496) sequences, respectively.

The presence of the *S*_1_, *S*_2_, *S*_3_, *S*_5_, *S*_7_, *S*_9_, *S*_10_, *S*_17_, *S*_20_, and *S*_24_-alleles in the heritage apples all confirmed using allele-specific primers (Broothaerts 2003), as well. Since the allele-specific primers FTC10 and FTC11 amplified the *S*_5_-allele unreliably, the amplicon from ʽKathreine’ using the ASPF3-F and S3/S5/S10-R primer pair was cloned and sequenced (PV693750) to confirm the identity of the allele; the fragment showed significant homology (*E* value: 5e-169) to the database apple *S*_5_-*RNases* with a single synonymous base substitution.

The *S*_9_- and *S*_46_-alleles (Long et al. 2010) are also very similar to each other and the sizes of the fragments amplified from these alleles using the consensus primers differ only by 1 bp. Therefore, the *S*_9_ allele-specific PCR products were sequenced to determine the specific alleles present in ʽChestnut Crab’, T45 and T184 (PV693744). In all three cases, the sequences were identical to the GenBank *S*_9_ sequence (MG598495). The *S*_31_-allele was cloned and sequenced from *Malus* × *purpurea* ʽRoyalty’ (PV693752) and 99.8% identical to the *S*_31_* Malus domestica* allele (DQ135990) with no amino acid replacements in the coded proteins.

The fragment lengths for ʽKing David’ using the ASPF3-F and EIIWPN-R primers indicated an *S* genotype of *S*_1_*S*_24_ that is further confirmed by negative *S*_9_ allele-specific and positive *S*_24_ allele-specific PCR results, which is incongruent with the commonly accepted *S*_1_*S*_9_ genotype for this variety (Morita et al. 2009).

### Ploidy levels

Because two heritage apples (ʽPink Parfait’ and ʽWaltana’) were shown to carry three *S*-alleles, their ploidy levels were determined using flow cytometry. The G_0_/G_1_ nuclei of the diploid control (ʽKing David’) showed a relative genome size of 71.86 with a CV value of 9.11. A smaller G_2_/M peak was also detected at X = 144.47 with a CV of 6.07. For the triploid control (ʽJonagold’), the G_0_/G_1_ nuclei measured 102.54 with a CV of 7.64, while the G_2_/M peak measured 203.42 with a CV of 5.88. These measurements confirmed that the genome size of the well-known triploid ʽJonagold’ is approximately one and a half times (143%) the genome size of the diploid ‘King David’ sample.

**Fig. 2:**
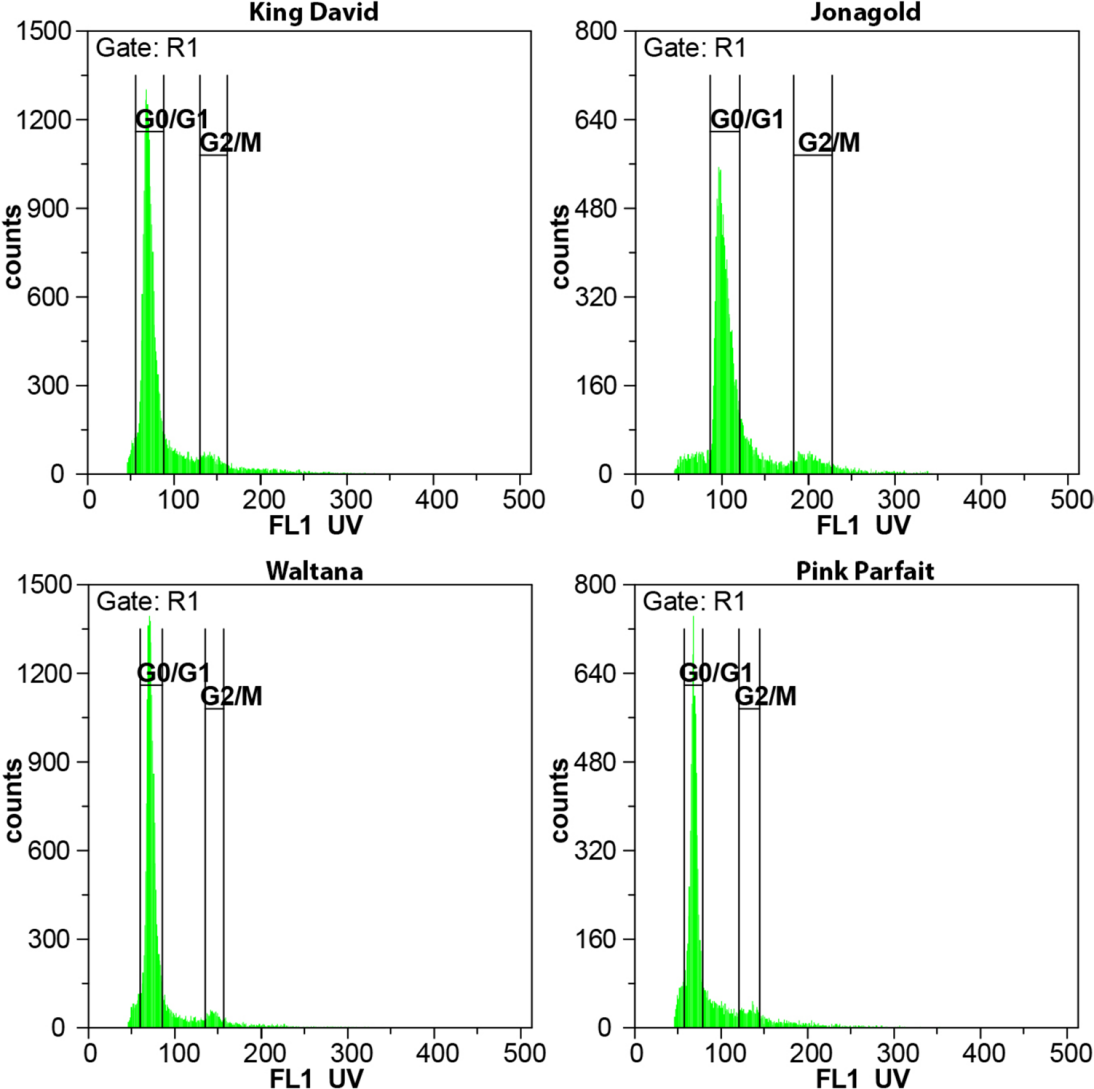
Histograms show the relative genome size of three different genotypes as determined by flow cytometry. The G_0_/G_1_ peak of the ‘Waltana’ genotype was detected at X = 71.65, which corresponds to the diploid control ʽKing David’ (G_0_/G_1_ peak at X = 70.46). The triploid control ʽJonagold’ showed a G_0_/G_1_ peak at X = 102.17. Secondary G_2_/M peaks were also observed in all samples: X = 140.41 (ʽKing David’), X = 202.83 (ʽJonagold’), and X = 141.23 (ʽWaltana’), confirming the diploid genome size of the ʽWaltana’ genotype

For ʽWaltana’, the G_0_/G_1_ peak was detected at 72.5 with a CV of 6.82, and the G_2_/M peak at 145.75 with a CV of 3.97. For ʽPink Parfait’, the G_0_/G_1_ peak was detected at 69.72 with a CV of 7.28, and the G_2_/M peak at 144.38 with a CV of 4.37. These values (101.88% and 97.98% relative to the diploid control) correspond to a diploid genome size, with deviations within 3% in both cases (Fig. 2).

### Genotyping for the red-flesh mutation

Using the method described by Espley et al. (2009) revealed that the promoter of the *MdMYB10* gene in all 80 new red-fleshed hybrids contained the six minisatellite repeats (R_6_) in either heterozygous (R_1_R_6_) or homozygous (R_6_R_6_) form (Table 3), confirming that all plants are type I red-fleshed mutants. This is not surprising as the mutant phenotype was evident from the red color of the first true leaves, making it useful for selection by breeders. The intensity of pigmentation in apple fruit flesh was not associated with the heterozygous or homozygous state of the R_6_ allele in the analyzed accessions as the red color intensity of R_1_R_6_ genotypes ranged from class 1 to 5 (Table 3).

**Table 3:** *S*-genotype and *MdMYB10* gene variants of red-fleshed hybrids and parental cultivars

Variety/hybrid	*MdMYB10*		Red color intensity	*S*-genotype	Parental cultivars
Red-fleshed parental cultivars					
*Malus* ʽGeneva’		R_1_R_6_	2	*S*_3_*S*_44_	
ʽAlmandin’		R_1_R_6_	4	S_1_*S*_3_	
*Malus* ʽRoberts’		R_6_R_6_	5	*S*_1_*S*_20_	
*Malus denboerii* ʽEvelyn’		R_6_R_6_	5	*S*_1_*S*_20_	
*Malus* × *purpurea* ʽRoyalty’		R_6_R_6_	5	*S*_17_*S*_31_	
White-fleshed parental cultivars					
ʽQuerina’		R_1_R_1_	–	*S*_3_*S*_9_	
ʽArtemisz’		R_1_R_1_	–	*S*_2_*S*_7_	
ʽRosmerta’		R_1_R_1_	–	*S*_9_*S*_10_	
E87		R_1_R_1_	–	*S_3_*S*_16b_	ʽReleika’ × Unknown
A376		R_1_R_1_	–	*S_2/10_*S*_28_	ʽGloster 69’ (*S*_28_*S*_?_) × ʽPrima’ (*S*_2_*S*_10_)
A56		R_1_R_1_	–	*S_2_*S*_7_	ʽPrima’ (*S*_2_*S*_10_) × Unknown
New red-fleshed hybrids					
T94		R_1_R_6_	4	*S*_2_*S*_20_	ʽArtemisz’ (*S*_2_*S*_7_) × *Malus denboerii* ʽEvelyn’ (*S*_1_*S*_20_)
T55		R_1_R_6_	3	*S*_2_*S*_20_	
X230		R_1_R_6_	3	*S*_7_*S*_20_	
X231		R_1_R_6_	3	*S*_7_*S*_20_	
X232		R_1_R_6_	4	*S*_2_*S*_20_	
X233		R_1_R_6_	3	*S*_2_*S*_20_	
X235		R_1_R_6_	4	*S*_2_*S*_20_	
X236		R_1_R_6_	4	*S*_7_*S*_20_	
X237		R_1_R_6_	4	*S*_2_*S*_20_	
X239		R_1_R_6_	3	*S*_1_*S*_2_	
X240		R_1_R_6_	4	*S*_2_*S*_20_	
X241		R_1_R_6_	3	*S*_2_*S*_20_	
X242		R_1_R_6_	4	*S*_2_*S*_20_	
X243		R_1_R_6_	3	*S*_2_*S*_20_	
X244		R_1_R_6_	4	*S*_1_*S*_2_	
T41		R_1_R_6_	3	*S*_1_*S*_7_	Supposed as ʽRosmerta’ (*S*_9_*S*_10_) proved as ʽArtemisz’ (*S*_2_*S*_7_) × *Malus denboerii* ʽEvelyn’ (*S*_1_*S*_20_)
T47		R_1_R_6_	4	*S*_2_*S*_20_	
X258		R_1_R_6_	4	*S*_20_*S*_44_	*Malus* ʽGeneva’ (*S*_3_*S*_44_) × *Malus* ʽRoberts’ (*S*_1_*S*_20_)
X266		R_1_R_6_	4	*S*_20_*S*_44_	
X147		R_1_R_6_	4	*S*_1_*S*_10_	Supposed as ʽQuerina’ (*S*_3_*S*_9_) proved as unknown (*S*_10_*S*_24_) × *Malus* ʽRoberts’ (*S*_1_*S*_20_)
X149		R_1_R_6_	2	*S*_1_*S*_24_	
X150		R_1_R_6_	4	*S*_1_*S*_10_	
X151		R_1_R_6_	4	*S*_10_*S*_20_	
X152		R_1_R_6_	4	*S*_10_*S*_20_	
X148		R_1_R_6_	2	*S*_9_*S*_20_	ʽQuerina’ (*S*_3_*S*_9_) × *Malus* ʽRoberts’ (*S*_1_*S*_20_)
X153		R_1_R_6_	3	*S*_9_*S*_20_	
X154		R_1_R_6_	3	*S*_1_*S*_3_	
X238		R_1_R_6_	3	*S*_1_*S*_3_	*Malus* ʽGeneva’ (*S*_3_*S*_44_) × ʽAlmandin’ (*S*_1_*S*_3_)
X272		R_1_R_6_	n.d	*S*_1_*S*_44_	
X273		R_1_R_6_	4	*S*_1_*S*_3_	
X274		R_1_R_6_	5	*S*_1_*S*_44_	
X275		R_1_R_6_	1	*S*_1_*S*_44_	
X276		R_6_R_6_	n.d	*S*_1_*S*_3_	
X277		R_1_R_6_	n.d	*S*_1_*S*_3_	
X278		R_1_R_6_	5	*S*_1_*S*_44_	
X279		R_6_R_6_	5	*S*_1_*S*_44_	
T97		R_1_R_6_	4	*S*_1_*S*_44_	
T98		R_1_R_6_	4	*S*_1_*S*_44_	
T40		R_1_R_6_	1	*S*_9_*S*_20_	ʽRosmerta’ (*S*_9_*S*_10_) × *Malus denboerii* ʽEvelyn’ (*S*_1_*S*_20_)
T45		R_1_R_6_	3	*S*_9_*S*_20_	
T53		R_1_R_6_	3	*S*_1_*S*_10_	
T255		R_1_R_6_	3	*S*_1_*S*_10_	
T264		R_1_R_6_	4	*S*_10_*S*_20_	
T138		R_1_R_6_	2	*S*_20_*S*_28_	A-376 (*S*_2/10_*S*_28_) × supposed as *Malus* × *purpurea* ʽRoyalty’ (*S*_17_*S*_31_) and proved as *Malus* ʽRoberts’ (*S*_1_*S*_20_)
T139		R_1_R_6_	2	*S*_20_*S*_28_	
T141		R_1_R_6_	2	*S*_20_*S*_28_	
T142		R_1_R_6_	3	*S*_1_*S*_28_	
T143		R_1_R_6_	2	*S*_1_*S*_28_	
X286		R_1_R_6_	4	*S*_1_*S*_3_	E-87 (*S*_3_*S*_16b_) × *Malus denboerii* ʽEvelyn’ (*S*_1_*S*_20_)
X287		R_1_R_6_	3	*S*_16b_*S*_20_	
X288		R_1_R_6_	4	*S*_1_*S*_16b_	
X289		R_1_R_6_	4	*S*_1_*S*_16b_	
X290		R_1_R_6_	4	*S*_1_*S*_16b_	
X291		R_1_R_6_	4	*S*_3_*S*_20_	
X292		R_1_R_6_	3	*S*_16b_*S*_20_	
X293		R_1_R_6_	4	*S*_16b_*S*_20_	
X294		R_1_R_6_	4	*S*_1_*S*_16b_	
X295		R_1_R_6_	3	*S*_1_*S*_16b_	
X296		R_1_R_6_	4	*S*_16b_*S*_20_	
X297		R_1_R_6_	4	*S*_1_*S*_16b_	
X298		R_1_R_6_	2	*S*_3_*S*_20_	
X299		R_1_R_6_	4	*S*_16b_*S*_20_	
X300		R_1_R_6_	4	*S*_1_*S*_3_	
X301		R_1_R_6_	3	*S*_1_*S*_3_	
X302		R_1_R_6_	2	*S*_3_*S*_20_	
X304		R_1_R_6_	4	*S*_3_*S*_20_	
X305		R_1_R_6_	4	*S*_16b_*S*_20_	
T96		R_1_R_6_	5	*S*_1_*S*_3_	
T164		R_1_R_6_	3	*S*_2_*S*_3_	A-56 (*S*_2_*S*_7_) × ʽAlmandin’ (*S*_1_*S*_3_)
T165		R_1_R_6_	2	*S*_1_*S*_7_	
X234		R_1_R_6_	4	*S*_1_*S*_28_	Unknown × *Malus denboerii* ʽEvelyn’ (*S*_1_*S*_20_)
X303		R_1_R_6_	5	*S*_1_*S*_8_	
T60		R_1_R_6_	2	*S*_7_*S*_20_	ʽHesztia’ (*S*_7_*S*_10_) × *Malus denboerii* ʽEvelyn’ (*S*_1_*S*_20_)
T123A		R_1_R_6_	2	*S*_2_*S*_3_	unknown
T163		R_1_R_6_	4	*S*_2_*S*_3_	A347 × ʽAlmandin’ (*S*_1_*S*_3_)
T184		R_1_R_6_	2	*S*_9_*S*_44_	ʽQuerina’ (*S*_3_*S*_9_) × *Malus* ʽGeneva’ (*S*_3_*S*_44_)
T249		R_1_R_6_	2	*S*_9_*S*_3_	*Malus* ʽGeneva’ (*S*_3_*S*_44_) × Unknown
X253		R_1_R_6_	3	*S*_1_*S*_2_	A-376 (*S*_2/10_*S*_28_) × *Malus* × *purpurea* ʽEleyi’
X283		R_1_R_6_	3	*S*_1_*S*_3_	ʽArtemisz’ (*S*_2_*S*_7_) × ʽAlmandin’ (*S*_1_*S*_3_)
J561		R_1_R_6_	2	*S*_9_*S*_20_	unknown
New non-red-fleshed cultivars and hybrids:					
ʽIsolda’		R_1_R_1_	–	*S*_16b_*S*_20_	
ʽVeritas’		R_1_R_1_	–	*S*_1_*S*_3_	
ʽBellona’		R_1_R_1_	–	*S*_3_*S*_8_	ʽFreedom’ × ʽFlorina’ (*S*_3_*S*_9_)
G103		R_1_R_1_	–	*S*_9_*S*_28_	ʽSnygold’ (Earligold) × ʽBaujade’
I142		R_1_R_1_	–	*S*_2_*S*_9_	
I233		R_1_R_1_	–	*S*_2_*S*_9_	

^*^S-genotypes inferred based on progeny *S*-genotypes; n.d., no data

## Discussion

### Methods and verification

Base pair-accurate amplicon sizes from consensus primer assays were determined and confirmed by allele-specific primers and sequencing for the* S*_1_, *S*_2_, *S*_3_, *S*_5_, *S*_6_, *S*_7_, *S*_9_, *S*_10_, *S*_16b_, *S*_17_, *S*_20_, *S*_24_, *S*_28_, *S*_31_, and *S*_44_-alleles (Table 1). The results obtained with the directly labeled forward primer were consistent and accurate to the base pair across numerous alleles and PCR reactions using DreamTaq™, Geneaid and Canvax HotBegan™ polymerases and master mixes, various reagents and DNA extracted with different kits.

This improvement was motivated by two key limitations identified in the assay developed by Larsen et al. (2016), which may have hindered the broader adoption. First, the forward primers incorporated an M13 tail that enabled indirect fluorescent labeling using a universal M13 primer (Schuelke 2000), an approach that can introduce systematic shifts in fragment size. Therefore, allele sizes reported in that study are not directly comparable with data obtained using directly labeled primers, even when identical consensus primers are used.

Fragment size differences between results using the M13 universal tail (Larsen et al. 2016) and the directly labeled forward primer in this study ranged from 15–18 bp (*S*_1_) to 25–27 bp (*S*_3_). The data indicates that size alterations were not solely attributable to the length of the M13 tail (19 bp). Larsen et al. (2016) reported fragments that were most frequently larger, and occasionally smaller, than the expected size by 1–3 bp. Size alterations between the directly labeled primers used in our study and the fragments amplified by M13 tail primer (Larsen et al. 2016) ranged from –3 to + 9 bp. Similarly, Mair et al. (2017) also observed larger deviations of 5–9 bp for several alleles. Regardless of the cause, deviations of this magnitude (≤ 12 bp) may introduce uncertainty in allele identification and hinder reliable comparisons across studies. Of the 36 alleles with known fragment sizes (Larson et al., 2016), 48 pairs of alleles may not be resolved if the accuracy is limited to 12 bp as reported for M13 tail primers.

In addition, the consensus primers themselves can generate fragments of identical or nearly identical size for certain allele pairs. Larsen et al. (2016) characterized those fragments by restriction digestion, an approach also employed by Alessandri et al. (2024) to differentiate amplicons generated with the PyCom C1F1 and PyCom C5R1 consensus primers. To avoid the high costs and labor associated with restriction enzymes, a PCR-only approach was developed to facilitate and streamline the workflow.

In breeding programs, unless there are multiple parental *S*-alleles present that generate fragments of identical length, no extra work is required as the PCR results in our approach are base pair accurate and thus unambiguous. If the specific alleles are present, the S16-R and S25-R reverse primers can be multiplexed with the S3/S5/S10-R reverse primers, as proposed by Larsen et al. (2016). Allele-specific primers are then only required to distinguish between fragments of identical size or to identify missing alleles. This approach significantly reduces the number of PCR reactions and associated costs. In population surveys where parentage is unknown, the consensus primers remain accurate at the base-pair level; however, care should be taken to confirm whether previously unidentified alleles with matching or similar amplicon sizes are present.

### Protocol minimal

Based on combined experimental and in silico analysis, we propose the following modifications to the protocol of Larsen et al. (2016) to resolve ambiguities and improve the reliability of allele discrimination (*S*_1_–*S*_47_), while minimizing the number of PCR reactions and eliminating the need for restriction enzyme digestion. For distinguishing between the *S*_9_ and *S*_46_ (1 bp difference) or the *S*_40_ and *S*_46_-alleles (2 bp difference), the MdS_46_SpF and MdS_46_SpR primer pairs (Long et al. 2010) can be used to detect the presence or absence of the *S*_46_-allele. In case of the *S*_9_ and *S*_40_-alleles (1 bp difference), the *S*_9_ allele-specific primers FTC154 and FTC155 (Broothaerts 2003) will not amplify the *S*_40_-allele. In the case of the *S*_2_ and S_23_-alleles (identical sizes), the OWB122 and OWB123 *S*_2_ allele-specific primers (Broothaerts 2003) will not amplify the *S*_23_-allele. To differentiate between the *S*_11_ and *S*_21_ (2 bp difference), the *S*_21_ and *S*_44_ (identical sizes), and the *S*_11_ and *S*_44_-alleles (2 bp difference), the MdS_21_SpF-MdS_21_SpR and MdS_44_SpF-MdS_44_SpR primer pairs (Long et al. 2010) can be used to detect the presence or absence of the *S*_21_ or *S*_44_ alleles. In case of the *S*_20_ and *S*_45_-alleles (1 bp difference), FTC141 and FTC142 primers (Broothaerts 2003) can be used as these will not amplify the *S*_45_-allele.

The *S*_6_/*S*_17_ and *S*_28_-alleles (2 bp difference) can be easily discerned using any of the available allele-specific primers. The fragments amplified from the *S*_6_ and *S*_17_-alleles using the consensus primers are identical in size (369 bp) but differ by five base substitutions (De Franceschi et al. 2018). The primer binding sites for the FTC141 and FTC142 primers (Broothaerts 2003), based on the full sequences (MG598492 and MG598499), are identical in both alleles making this primer pair incapable of discriminating between these two alleles. The fragments amplified with the consensus primers can also be cloned and sequenced for critical applications or the *S*_17_ amplicons can be identified based on a unique restriction site for *MluI* at 255 bp (providing fragments of 255 and 114 bp). However, the functional distinction between *S*_6_ and *S*_17_ has not yet been demonstrated (De Franceschi et al. 2018), and an increased *S*-genotype resolution is only warranted if the two alleles are functionally distinct.

Any other *S*-allele combinations published by Larsen et al. (2016) differ by at least 3 bp in length and discerning between these should be straightforward using the electropherograms of directly labeled base pair accurate amplicons even without prior knowledge of possible *S*-alleles present in the samples.

### Genotyping the new hybrids

In order to *S*-genotype the new red-fleshed hybrids, the 11 parental cultivars were to be genotyped first. However, plant material was unavailable for the parents E87, A376, and A56. E87 is an open pollinated seedling of ʽReleika’, itself with an undetermined *S*-genotype, A376 is a cross between ʽGloster 69’ (*S*_28_*S*_?_, Morita et al. 2009) and ʽPrima’ (*S*_2_*S*_10_, Tóth et al. 2012) and A56 is an open pollinated seedling of ʽPrima’. Based on these data, the expected *S*-allele pool of the new red-fleshed hybrids was determined as *S*_1_–*S*_3_, *S*_7_, *S*_9_, *S*_10_, *S*_17_, *S*_20_, *S*_31_, and *S*_44_. During the analysis, the *S*_8_, *S*_16_, and *S*_28_-alleles were also detected. These results and the pedigree records helped assign the *S*_3_*S*_16b_ and *S*_2_*S*_7_ genotypes to E87 and A56, respectively. The results for A376 were inconclusive as all its progenies inherited the *S*_28_-allele (transferred from ʽGloster 69’), making the *S*-genotype of A376 either *S*_2_*S*_28_ or *S*_10_*S*_28_.

Results of the new red-fleshed hybrids also pointed to procedural errors during pollination and/or seed handling in some cases. T41 and T47 were supposed to be crosses between ʽRosmerta’ (*S*_9_*S*_10_) and *Malus denboerii* ʽEvelyn’ (*S*_1_*S*_20_). However, their *S*-genotypes are *S*_1_*S*_7_ for T41 and *S*_2_*S*_20_ for T47. ʽArtemisz’ (*S*_2_*S*_7_) was determined as a seed parent in both cases. T138–T143 were all recorded as crosses between A376 (*S*_2/10_*S*_28_) and *Malus purpurea* ʽRoyalty’ (*S*_17_*S*_31_); however, none of these hybrids carry the *S*_17_ or *S*_31_ alleles; the pollinating parent was determined to be *Malus* ʽRoberts’ (*S*_1_*S*_20_) based on *S*-alleles present in all five hybrids. The X147–154 hybrids were supposed to be crosses between ʽQuerina’ (*S*_3_*S*_9_) and *Malus* ʽRoberts’ (*S*_1_*S*_20_); however, neither of the hybrids carry the *S*_3_- or *S*_9_-alleles. Instead, the second allele of X149 is *S*_24_ and for the others, it is *S*_10_, and the source of those alleles could not be identified.

### *S*-genotyping of American heritage apples

In the American heritage apples, two alleles were amplified in seven varieties each supporting their diploid status; however, in ʽPink Parfait’ and ʽWaltana’, the consensus primers amplified three alleles. The presence of all detected alleles in the heritage apples was confirmed with allele-specific primers (Broothaerts 2003; Long et al. 2010) and the *S*_1_, *S*_10_, and *S*_17_-alleles amplified from ʽWaltana’ were also cloned and sequenced. The *S*_1_ and *S*_10_ amplicons were 99.8% to 100% identical to the GenBank sequences; the *S*_17_ nucleotide sequence was 97.3% identical to the GenBank sequence with one asparagine to lysine conservative replacement resulting in a 98.5% match in the encoded protein sequence. Ploidy levels were also confirmed for all heritage apples using flow cytometry, and all were determined to be diploid. Pinpointing the exact phenomenon behind the results requires further, more focused research. A segmental duplication on chromosome 17 involving one *S*-allele may explain these results, similar to mutations reported in *Pyrus* (Mase et al. 2014; Nishio et al. 2024). The occurrence of this putative mutation in two genotypes can be attributed to the shared origin of the ʽWaltana’ and ʽPink Parfait’ varieties (Gruenfeld and Hill 2025).

### *S*-allele frequencies

**Fig. 3:**
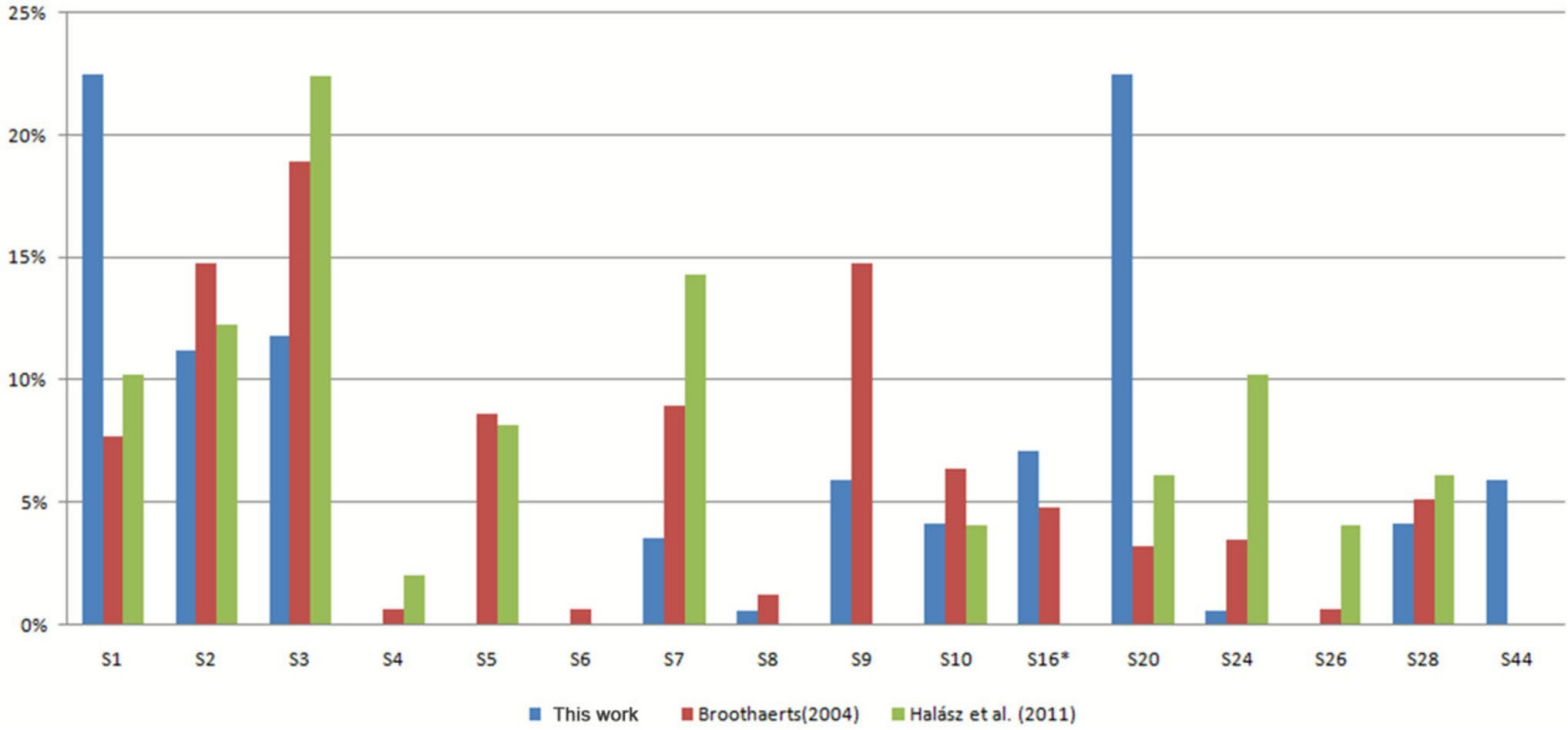
*S*-allele frequency distribution among the new red-fleshed hybrids *S*-genotyped in this work (blue), among the most popular commercial cultivars worldwide (red, Broothaerts, 2004) and among the old Hungarian cultivars and landraces (green, Halász et al. 2011). Results under *S*_16b_ include *S*_22_, *S*_23_ and *S*_25_ from Broothaerts (2004) work based on these alleles coding for the same protein as *S*_16b_ (Matsumoto and Furusawa, 2005)

The high frequency (23%) of *S*_1_- and *S*_20_-alleles (Fig. 3) aligns with the extensive use of *Malus* ʽRoberts’ and *Malus denboerii* ʽEvelyn’ as R6 donors. In addition, each of these alleles appeared in other parental cultivars (*S*_1_ in ʽAlmandin’ and *S*_20_ in ʽIsolda’). Although the *S*-allele composition of the new hybrids is predetermined due to the small number of parental cultivars used, the *S*_2_- and *S*_3_-alleles are still present in 11–12% of the new hybrids. This ratio is much lower than in other populations; the worldwide overrepresentation of these two alleles was previously linked to the overuse of disease-resistant accessions as parents (Nybom et al. 2008; Alessandri et al. 2024). Of the 11 parental cultivars, six are resistant to apple scab, powdery mildew and/or fire blight; three of these carry the *S*_2_- and another three the *S*_3_-allele, yet both can be considered underrepresented compared to other populations (Broothaerts et al. 2004; Nybom et al. 2008; Halász et al. 2011; Larsen et al. 2016; De Franceschi et al. 2018; Alessandri et al. 2024). Compared to traditional Hungarian varieties (Halász et al. 2011), the *S*_7_- and *S*_24_-alleles are significantly less common and the *S*_5_ is completely absent in the new hybrids. Although the *S*_24_-allele was not present in the parents used for crossbreeding, it is relatively common in Hungarian germplasm, and several cultivars carrying it were present in the orchard where pollination work was done, suggesting these as the likely source of the allele in the X149 hybrid.

The characteristic differences in allele frequencies suggest that the hybrids could serve as promising accessions for breeding programs aimed at increasing genetic diversity in pathogen-resistant cultivars.

## Conclusions

An optimized high-throughput *S*-genotyping protocol for apple is reported here, providing base pair-accurate allele identification without reliance on restriction enzyme digestion. Our protocol facilitates direct comparison of data across studies. We applied this method to 80 novel red-fleshed and six white-fleshed apple hybrids, as well as their parental cultivars, revealing 16 distinct *S*-alleles and resolving several methodological inconsistencies reported in previous studies. All red-fleshed hybrids were confirmed to carry the R_6_ allele of the *MdMYB10* gene, validating their type I red-fleshed phenotype. The *S*-genotyping results also uncovered discrepancies in pedigree records and pollination procedures, highlighting the utility of molecular markers in breeding program quality control. Ploidy analysis of heritage apples confirmed diploidy despite the presence of three *S*-alleles, suggesting possible segmental duplications requiring further investigation. The observed *S* allele frequencies differed from those in traditional Hungarian and international germplasm, indicating that these hybrids may contribute to enhancing genetic diversity in disease-resistant apple breeding. Overall, the standardized, high-resolution and efficient genotyping approach and the characterized hybrids provide valuable tools and resources for future apple breeding and germplasm management.

## Supplementary Information

Below is the link to the electronic supplementary material.

Supplementary file1 (DOCX 20 KB)

## Data Availability

A total of 9 sequences were submitted to the NCBI GenBank database under the accession numbers of PV693744–PV693752. The datasets used and/or analyzed during the current study are available from the corresponding author on reasonable request.

## Declarations

### Generative AI and AI-assisted technologies in the writing process

During the preparation of this work, the authors used M365 copilot only to improve readability and flow of the text. After using this tool, the authors reviewed and edited the content as needed and take full responsibility for the content of the published article.
