# Supplementary material for: Direct fluorescent S-genotyping reveals genetic diversity and pedigree inconsistencies in red-fleshed apple hybrids and American heritage varieties
Source: Planta. 2026 Jan 6;263(2):48. doi: 10.1007/s00425-025-04918-4 (PMC12775039; doi:10.1007/s00425-025-04918-4)
Supplement: Supplementary file 1 — Supplementary file1 (DOCX 20 KB) [file 425_2025_4918_MOESM1_ESM.docx]

Supplementary Table S1. List of primers used in this study for *S*-genotyping and their characteristics

| Primer name | Sequence 5'-3' | Amplicon size (bp) | Annealing temperature (^o^C) | Reference |
| --- | --- | --- | --- | --- |
| ASPF3-F | CAATTTACGCAGCARTATCAG | Variable |  | Kim et al., 2006 |
| EIIWPN-R | ACGTTYGGCCAAATAATWDCC | Variable | 62-> 53 | Larsen et al., 2016 |
| S3/S5/S10-R | TGTTTTGAATYGAAAATTARTTAGGAGT | Variable | 58 | Larsen et al., 2016 |
| S16-R | TGGAAGAGGGCAATTTTGG |  | 58 | Larsen et al., 2016 |
| S25-R | TGAAAATGGCTGAAAAACTTTG |  | 58 | Larsen et al., 2016 |
| S8-F | TACGATTATTTTCAATTTACGCTT | 160 | 58 | Larsen et al., 2016 |
| S8-R | ATTTAAGGTTGTTTCTTTGCAATAC |  |  | Larsen et al., 2016 |
| FTC168 | ATATTGTAAGGCACCGCCATATCAT | 530 | 60 | Broothaerts, 2003 |
| FTC169 | GGTTCTGTATTGGGGAAGACGCACAA |  |  | Broothaerts, 2003 |
| OWB122 | GTTCAAACGTGACTTATGCG | 449 | 60 | Broothaerts, 2003 |
| OWB123 | GGTTTGGTTCCTTACCATGG |  |  | Broothaerts, 2003 |
| FTC141 | ATCAGCCGGCTGTCTGCCACTC | 850 (*S*_6_, *S*_17_), 920 (*S*_20_) | 60 | Broothaerts, 2003 |
| FTC142 | AGCCGTGCTCTTAATACTGAATAC |  |  | Broothaerts, 2003 |
| FTC143 | ACTCGAATGGACATGACCCAGT | 302 | 60 | Broothaerts, 2003 |
| FTC144 | TGTCGTTCATTATTGTGGGATGTC |  |  | Broothaerts, 2003 |
| FTC154 | CAGCCGGCTGTCTGCCACTT | 343 | 60 | Broothaerts, 2003 |
| FTC155 | CGGTTCGATCGAGTACGTTG |  |  | Broothaerts, 2003 |
| FTC231 | AAATATTGCAACGCACAGCA | 580 | 60 | Broothaerts, 2003 |
| FTC232 | TTGAGAGGATTTCAGAGATG |  |  | Broothaerts, 2003 |
| FTC229 | TCTGGGAAAGAGAGTGGCTC | 304 | 60 | Broothaerts, 2003 |
| FTC230 | TTTATGAACTTCGTTAAGTCTC |  |  | Broothaerts, 2003 |
| FTC177 | CAAACGATAACAAATCTTAC | 500 | 61-> 54 | Broothaerts, 2003 |
| FTC226 | TATATGGAAATCACCATTCG |  |  | Broothaerts, 2003 |
| FTC10 | CAAACATGGCACCTGTGGGTCTCC | 346 | 60 | Broothaerts, 2003 |
| FTC11 | TAATAATGGATATCATTGGTAGG |  |  | Broothaerts, 2003 |
| FTC12 | CCAAACGTACTCAATCGAAG | 209 | 60 | Broothaerts, 2003 |
| FTC228 | ATGTCGTCCCGTGTCCTGAATC |  |  | Broothaerts, 2003 |
| MdS11SpF | AAATATTGCAAGGCGCCGC | 678 | 63 | Long et al., 2010 |
| MdS11SpR | TTTCAATATCTACCAGTCTCCGGC |  |  | Long et al., 2010 |
| MdS21SpF | AAGTAATTGCCCGATAAGGAACATA | 584 | 63 | Long et al., 2010 |
| MdS21SpR | AGTTTATGAAATGTTCTCCGCTGTA |  |  | Long et al., 2010 |
| MdS44SpF | GCATGGTAGGACCTGACCCAAGTA | 561 | 63 | Long et al., 2010 |
| MdS44SpR | TCTCAACCAATTGAGTCGTCGTACC |  |  | Long et al., 2010 |

Supplementary Table S2. PCR programs used for *S*-allele amplification

| **Program** | **Primer pairs** | **Initial step** | **Cycles** | **Denaturation** | **Annealing** | **Extension** | **Final step** | **Notes** |
| --- | --- | --- | --- | --- | --- | --- | --- | --- |
| A | ASPF3-F / EIIWPN-R | 94 °C  2 min | 18 touchdown + 20 regular | 94 °C, 1 min | Touchdown:  62 °C decreasing by 0.5 °C per cycle; then 53 °C | 72 °C, 1 min | 72 °C 10 min  4 °C hold | Touchdown phase followed by regular cycles |
| A (modified) | FCT177 / FTC226 | 94 °C  2 min | 14 touchdown + 20 regular | 94 °C, 1 min | Touchdown starts at 61 °C; regular cycles at 54 °C | 72 °C, 1 min | 72 °C, 10 min  4 °C hold | Same as A with adjusted temperatures and cycle numbers |
| B | ASPF3-F / S3-, S5-, S10-R; S16-R; S25-R; S8F / S8R | 94 °C  2 min | 33 | 94 °C, 20 s | 58 °C, 20 s | 72 °C, 30 s | 72 °C, 10 min  4 °C hold | — |
| C | Broothaerts (2003) allele-specific primers | 94 °C  2 min | 30 | 94 °C, 20 s | 60 °C, 20 s | 72 °C, 50 s | 72 °C, 7 min  4 °C hold | For MdS11SpF/R, MdS21SpF/R, MdS44SpF/R: annealing at 63 °C |

Hot-start note: For reactions using hot-start polymerases, the initial denaturation was 95 °C for 10 min, and denaturation during cycles was performed at 95 °C; all other steps were identical to the programs described above.
